# Single Chain Variable Fragment Against Aβ Expressed in Baculovirus Inhibits Abeta Fibril Elongation and Promotes its Disaggregation

**DOI:** 10.1371/journal.pone.0124736

**Published:** 2015-04-28

**Authors:** Ying Zhang, Hai-Qiang Yang, Fang Fang, Lin-Lin Song, Yue-Ying Jiao, He Wang, Xiang-Lei Peng, Yan-Peng Zheng, Jun Wang, Jin-Sheng He, Tao Hung

**Affiliations:** 1 College of Life Sciences and Bioengineering, Beijing Jiaotong University, Beijing, China; 2 Institute for Viral Disease Control and Prevention, China CDC, Beijing, China; Van Andel Institute, UNITED STATES

## Abstract

Alzheimer’s disease (AD) is the most common form of age-related dementia, and the most urgent problem is that it is currently incurable. Amyloid-β (Aβ) peptide is believed to play a major role in the pathogenesis of AD. We previously reported that an Aβ N-terminal amino acid targeting monoclonal antibody (MAb), A8, inhibits Aβ fibril formation and has potential as an immunotherapy for AD based on a mouse model. To further study the underlying mechanisms, we tested our hypothesis that the single chain fragment variable (scFv) without the Fc fragment is capable of regulating either Aβ aggregation or disaggregation *in vitro*. Here, a model of cell-free Aβ “on-pathway” aggregation was established and identified using PCR, Western blot, ELISA, transmission electron microscopy (TEM) and thioflavin T (ThT) binding analyses. His-tagged A8 scFvs was cloned and solubly expressed in baculovirus. Our data demonstrated that the Ni-NTA agarose affinity-purified A8 scFv inhibited the forward reaction of “on-pathway” aggregation and Aβ fibril maturation. The effect of A8 scFv on Aβ fibrillogenesis was markedly more significant when administered at the start of the Aβ folding reaction. Furthermore, the results also showed that pre-formed Aβ fibrils could be disaggregated via incubation with purified A8 scFv, which suggested that A8 scFv is involved in the reverse reaction of Aβ aggregation. Therefore, A8 scFv was capable of both inhibiting fibrillogenesis and disaggregating matured fibrils. Our present study provides valuable insight into the regulators of ultrastructural dynamics of cell-free “on-pathway” Aβ aggregation and will assist in the development of therapeutic strategies for AD.

## Introduction

Alzheimer’s disease (AD) is the most common and refractory neurodegenerative disease and has led to a huge socioeconomic and humanistic problem [1]. The pathological features of AD include progressive neuronal loss, the formation of beta amyloid (Aβ) plaques called senile plaques (SPs), and the accumulation of tau protein neurofibrillary tangles [2]. Aβ is generated from amyloid β precursor protein (APP) through its cleavage by β- and γ- secretases in the amyloidogenesis pathway and is expressed normally and ubiquitously as a peptide consisting of 39–43 residues during the trafficking and turnover process [2,3]. During the process of fibrillogenesis, Aβ forms oligomers, protofibrils and ultimately fibrils that are deposited in the extracellular area of the brain as “amyloid plaques” [4]. Interestingly, Aβ fibrillogenesis may directly contribute to the initiation and progression of AD pathogenesis according to the “amyloid cascade hypothesis” [5], although the mechanisms of AD pathogenesis are poorly understood. Therefore, studies investigating Aβ polymerization at the molecular level, the ultrastructural characteristics of Aβ fibrils, and the effects of endogenous and exogenous intervention in Aβ fibrillation will facilitate the design of Aβ aggregation inhibitors.

To date, the inhibition and regulation mechanisms of Aβ aggregation are not well understood. All of the symptomatic treatments currently available for AD, including the cholinesterase inhibitors donepezil, galantamine, and rivastigmine and the noncompetitive N-methyl-D-aspartate receptor antagonist memantine, do not affect the underlying amyloid deposition and neurodegenerative processes [6]. Therefore, there is an urgent need to develop novel drugs that could slow down the Aβ aggregation-based neurodegenerative process and exert a disease-modifying effect.

The process of Aβ aggregation can be divided into two categories, “off-pathway” and “on-pathway”, but there is no fibril formation in the “off-pathway” model [7]. In the “on-pathway” model, the entire reaction is composed of two steps, which can be directly analyzed under transmission electron microscopy (TEM): (i) a slow nucleation step or “lag phase” and (ii) a rapid fibril elongation step [7,8,9]. However, the experimental model of “on-pathway” Aβ aggregation is difficult to establish within cells because of the pre-aggregates that contain β-sheets [10] (which may be due to partially folded monomers [11]). TEM can be used to exclude the pre-aggregates of Aβ to record and confirm the *de novo* “on-pathway” model in a cell-free system.

On-pathway Aβ aggregation can be used as a peptide aging model to investigate the mechanisms of Aβ fibrillogenesis and to develop inhibitors of Aβ aggregation on the basis of the length and sometimes the diameter of the Aβ fibrils. On the one hand, monomer nucleation may represent an early stage of Aβ aggregation in AD, and the “window” around the nucleation period appears to be the correct target for drug design and therapy in the early stages. On the other hand, protofibril elongation and amyloid plaque formation represent, respectively, the pathology in the middle and late stages of AD, and a method for degrading fibrils may provide new insights toward therapies for late-stage AD. However, it is poorly understood how the fibrils are degraded in a reverse reaction of Aβ disaggregation.

The results of Aβ protein analysis also provided clues to the nature of “self-associating” assembly. In SPs, the major component is Aβ42, whereas Aβ40 is preferentially found in cerebral amyloid angiopathy (CAA). The determinant of aggregation of Aβ42 is distinctly different from that of Aβ40 [7]. Generally, in Aβ42, residues 18–26 and 31–42 form β-strands, whereas in Aβ40, residues 12–24 and 30–40 form parallel β-sheets [7]. The C terminal amino acids appear to be critical for Aβ monomer nucleation, raising questions regarding how N-terminus targeted therapies attenuate the Aβ load in mouse models [12].

As we previously reported, a strain of a monoclonal antibody against Aβ42 oligomers (designated as A8 [13,14]) was prepared and employed as a passive immunotherapy approach to treat SAMP8 (senescence-accelerated mouse sub-line P8) mice, an animal model of AD. A8 was shown to inhibit Aβ-derived cell toxicity and suppress Aβ aggregation to an effective degree *in vitro*; however, the mechanism by which this is achieved is not known [13]. This N terminus-targeted MAb has been reported to have potential anti-Aβ aggregation activity, although the C terminus may be the determinant of nucleation. However, whole antibodies are unwieldy and undergo complex biogenesis, and their large genes are not suitable for efficient genetic transfer with vectors [15]. It is unclear how A8 interrupts Aβ fibrillation and whether the Fc fragment is required for the anti-aggregation effect. Antigen-binding fragments of antibodies can be refolded from a denatured state with the recovery of their specific binding ability [16]. One of the smallest fragments that contains a complete binding site is called the single chain fragment variable (scFv) [17,18], which consists of a heterodimer of the VH and VL domains [19]. If the variable region of the heavy and light chains is sufficient to regulate Aβ self-association, the entire molecule would decrease in size and the inflammation caused by Fc activation may be avoided in immunotherapy.

In this study, based on the sequence of the variable region of MAb A8, A8-derived recombinant scFv gene fragments were assembled via SOE-PCR and separately expressed in baculovirus systems. The parameters were concurrently optimized and the cell-free model of Aβ aggregation was established in a modified borate buffer. Using this model, our results showed clear ultrastructural characteristics of Aβ aggregation morphology under TEM, which can be used to determine the efficacy of inhibitors. Furthermore, anti-Aβ scFvs were used to regulate the kinetics of Aβ aggregation and disassembly at different stages. Our data showed that a scFv without the Fc fragment was capable of inhibiting Aβ aggregation and fibril elongation. Notably, the effect of this scFv was substantially significant when administered beginning at the initiation of the assembly reaction. Additionally, mature Aβ fibrils can be disaggregated by an anti-Aβ scFv targeting N-terminal amino acids 1–6. This study is the first to analyze the bidirectional regulation of the ultrastructural kinetics of on-pathway Aβ aggregation via anti-Aβ scFv in a cell-free system and provides an effective model of and insight into the control and regulation of Aβ on-pathway assembly.

## Materials and Methods

### Protein expression system

Baculovirus package pFastBac vector, MAX efficiency DH10Bac and Cellfectin II reagent were included in the Bac-to-Bac Baculovirus Expression System kit (Life Technology, Carlsbad, CA, USA). The Sf9 cell line was purchased from ATCC (CRL 1711). The prokaryotic expression vector pET-30a (+) and the recipient *Escherichia coli* (*E*.*coli*) BL21 cells were obtained from Novagen.

### Reagents and antibodies

Aβ1–42 peptide (95% purity) (DAEFR HDSGY EVHHQ KLVFF AEDVG SNKGA IIGLM VGGVV IA) was synthesized by the Shanghai Sangon Biological Engineering Technology and Services Company (Shanghai, China); 1,1,1,3,3,3-hexafluoro-2-propanol (HFIP) was purchased from Fluka (Fluka Corporation, Everett, WA, USA). Uranyl acetate and dimethyl sulfoxide (DMSO) were purchased from Sigma-Aldrich (Sigma-Aldrich, St. Louis, MO, USA). The A8 monoclonal antibody was developed in our lab as described previously [13,14]. The rabbit polyclonal Ab to the 6× His tag (HRP) was purchased from Abcam (Abcam Company, Cambridge, UK), and the Sf-900 II SFM medium was purchased from Invitrogen (Life Technology, Carlsbad, CA, USA).

### Amplification and construction of anti-Aβ scFv gene fragments

RNA was extracted from A8 [14] hybridoma cells, and cDNA was obtained via subsequent reverse transcription-PCR. VH and VL fragments were amplified through 5’RACE and sequenced as described previously [20]. Based on the sequence of the variant region of Mab A8 [20], the VL, (G_4_S)_3_, and VH regions were joined through gene splicing via overlap extension PCR (SOE PCR) and cloned into the pMD-18T vector for sequencing. The primers used for VL-(G_4_S)_3_)-VH and VH-(G_4_S)_3_-VL are listed in Table 1.

**Table 1:** The primers used for VL-(G_4_S)_3_-VH and VH-(G_4_S)_3_-VL amplification.

Primer ID	Sequences
VL F:	5’-AGCGGATCCGATGTTTTGATGACCCAA-3’
VL R:	5’-CGAGCCTCCACCGCCTGAGCCACCTCCGCCAGAACCGCCTCCACCTTTCAGCTCCAGCTTGGT-3’
VH F:	5’-GGTGGAGGCGGTTCTGGCGGAGGTGGCTCAGGCGGTGGAGGCTCGCAAGTTACTCTAAAAGAG-3’
VH R:	5’-ATACTCGAGTGAGGAGACTGTGAGAGT-3’
VH F2:	5’-CGCGGATCCACAAGTTACTCTAAAAGAG-3’
VH R2:	5’-CGAGCCTCCACCGCCTGAGCCACCTCCGCCAGAACCGCCTCCACCTGAGGAGACTGTGAGAGT-3’
VL F2:	5’-GGTGGAGGCGGTTCTGGCGGAGGTGGCTCAGGCGGTGGAGGCTCGGATGTTTTGATGACCCAA-3’
VL R2:	5’-ATACTCGAGCTTTCAGCTCCAGCTTGGT-3’

### Construction and identification of rBacmid containing anti-Aβ scFv

Two orientations of scFvs were expressed in *E*. *coli* in our preliminary experiments, and the orientation with higher expression level was selected for expression in the baculovirus system. The versions of scFvs were summarized in Table 2. N-terminal and C-terminal His-tags were added to the VL-(G_4_S)_3_-VH orientation (Table 2), in which the *Bam*HI/*Xho*I sites were introduced through PCR. The primers were designed as follows: for VL-(G_4_S)_3_-VH-His, 5’-GCC GGA TCC ATG GAT GTT TTG ATG ACC CAA-3’ was used as the forward primer and 5’-CCG CTC GAG ACT GTG ATG GTG ATG GTG ATG TGA GGA GAC TGT GAG AGT-3’ was used as the reverse primer; for His-VL-(G_4_S)_3_-VH, 5’-GCC GGA TCC ATG CAT CAC CAT CAC CAT CAC GAT GTT TTG ATG ACC CAA-3’ was used as the forward primer and 5’-CCG CTC GAG ACT TGA GGA GAC TGT GAG AGT-3’ was used as the reverse primer. We introduced *Bam*HI sites into the forward primers and *Xho*I into the reverse primers. PCR was performed under the following conditions: initial denaturation, 5 min at 94°C; denaturation, 30 sec at 94°C; annealing, 30 sec at 55°C; extension, 1 min at 72°C; and final extension, 10 min at 72°C, 30 cycles. Agarose gel electrophoresis was performed to confirm the size of the PCR products.

**Table 2:** Summary of scFvs used in present study.

Expression systems	scFvs Orientations	His-tag location	Purification methods
*Escherichia coli*	VL-(G_4_S)_3_-VH (VL-VH), and VH-(G_4_S)_3_-VL (VH-VL)	N-terminal, on the prokaryotic expression vector pET-30a (+)	Ni-NTA column
Baculovirus	VL-(G_4_S)_3_-VH	N-terminal or C-terminal His-tag, His-VL-(G_4_S)_3_-VH and VL-(G_4_S)_3_-VH-His, introduced by PCR	Ni-NTA column

The anti-Aβ-scFv rBacmid was constructed and the anti-Aβ scFvs were expressed using the Bac-to-Bac Baculovirus Expression System (Invitrogen by Life Technologies, Carlsbad, CA, USA) according to the manufacturer’s instructions. In brief, His-VL-(G_4_S)_3_-VH and VL-(G_4_S)_3_-VH-His were inserted into pFastBac1 to construct the recombinant plasmids pFastBac 1-His-VL-(G_4_S)_3_-VH and pFastBac 1-VL-(G_4_S)_3_-VH-His, respectively. Then, the recombinant plasmids were transformed into DH10Bac *E*. *coli* for transposition into the bacmid. The cells were grown on solid medium for 48 hours at 37°C, and white colonies were cultured overnight to create a mini preparation of bacmid DNA. The identification of bacmid DNA was performed using PCR according to the manufacturer’s instructions. The pUC/M13 forward (5’-GTT TTC CCA GTC ACG AC-3’) and the pUC/M13 reverse primers (5’-CAG GAA ACA GCT ATG AC-3’) were provided by Invitrogen in the Bac-to-Bac Baculovirus Expression System kit. Agarose gel electrophoresis was performed for further analysis of the PCR products.

### Generation of the recombinant baculovirus stock

Sf9 cells, a clonal isolate of *Spodoptera frugiperda* Sf21 cells (IPLB-SF21-AE), were grown in T25 cell culture flasks with complete growth medium (Sf-900 II SFM, Invitrogen, Carlsbad, CA, USA) at 27°C without CO_2_, and the cells were diluted 1:3 when they covered the bottom of the flask. The cells in the logarithmic growth phase were transfected with the recombinant baculovirus bacmid DNA encoding anti-Aβ scFv using the Cellfectin reagent (Invitrogen, Carlsbad, CA, USA) as described by the manufacturer. The supernatant containing recombinant budded viruses, designated P1, were harvested 72 h after infection and centrifuged at 500 × g for 5 min to remove cellular debris. Generally, the P1 viruses were amplified through three consecutive rounds of Sf9 cell infection at a high multiplicity of infection (MOI, 20 plaque-forming units per cell) to obtain the P3 virus.

### Expression and purification of anti-Aβ scFv from baculovirus

The expression of His-VL-(G_4_S)_3_-VH and VL-(G_4_S)_3_-VH-His was performed via infection of approximately 8×10^5^ Sf9 cells using the third generation (P3) of the recombinant viruses, and the cellular and medium fractions of transfected cells were harvested at 72 h. After the cells were harvested and washed with phosphate-buffered saline (PBS), the whole cell protein was extracted with lysis buffer (50 mM NaH_2_PO_4_, 300 mM NaCl, 10 mM imidazole, pH 8.0). After centrifugation at 3,000 rpm for 5 min, the supernatant was stored at -20°C.

The His-tag fusion proteins were purified using Ni-NTA agarose (QIAGEN). To the cleared lysate, we added 200 μl of 50% Ni-NTA slurry per 4 ml of cleared lysate, which was mixed gently by shaking (200 rpm) at 4°C overnight. The lysate-Ni-NTA mixture was loaded into a column in which the outlet was initially capped; the outlet cap was then removed, and the column flow-through (FL) fraction was collected. After the FL was drained, we washed the mixture twice with 1 ml of wash buffer (50 mM NaH_2_PO_4_, 300 mM NaCl, 20 mM imidazole, pH 8.0) and collected the wash fractions. Finally, we eluted the protein four times with 300 μl of elution buffer (50 mM NaH_2_PO_4_, 300 mM NaCl, 250 mM imidazole, pH 8.0). The eluates were collected in four tubes and analyzed via Western blot (the primary antibody was a monoclonal rabbit anti-His Ab, 1:1,000, Abcam, Cambridge, UK). The experiments were repeated three times, and the SDS-PAGE gels were stained with Coomassie brilliant blue and probed with the rabbit anti-His tag antibody [13].

### Indirect ELISA

In the indirect enzyme-linked immunosorbent assay (ELISA), 96-well microtiter ELISA plates were coated with 200 ng/well of Aβ42 oligomer mixture in carbonate-bicarbonate coating buffer (0.015 M Na_2_CO_3_, 0.035 M NaHCO_3_, pH 9.6) at 4°C overnight. The coating buffer was then replaced with 100 μl of blocking buffer (137 mM NaCl, 2.7 mM KCl, 10 mM Na_2_HPO_4_, 2 mM KH_2_PO_4_, pH 7.0 containing 5% BSA), and the plates were incubated at 37°C for 2 h. After draining, the plates were washed three times with wash buffer containing 137 mM NaCl, 2.7 mM KCl, 10 mM Na_2_HPO_4_, 2 mM KH_2_PO_4_, pH 7.0, 0.05% Tween-20 (pH 7.0). His-VL-(G_4_S)_3_-VH (1.39 mg/ml) and VL-(G_4_S)_3_-VH-His (1.19 mg/ml) (100 μl/well) were added and incubated at 37°C for 2 h (1 μg/ml of A8 was used as a positive control). The plates were then washed three times as described previously. Then, an HRP-labeled second antibody (1:5,000 dilution, 100 μl/well) was added and incubated at 37°C for 1 h. After washing three times, 3,3’,5,5’-tetramethylbenzidine (TMB, Sigma-Aldrich) peroxidase substrate (100 μl/well) was added and incubated at room temperature for 15 min. Then, stop solution (2 M H_2_SO_4_, 50 μl/well) was added, and the absorbance was read at 450 nm using a Sunrise Plate reader (Tecan, Männedorf, Switzerland). The experiments were repeated at least three times.

### Dot blot

For this experiment, 3 μl of Aβ42 oligomers (0.5 mg/ml) were added to a nitrocellulose membrane, and the dots were then allowed to dry. Each blot was blocked with 5% skim milk in TBST (20 mM Tris-HCl, 150 mM NaCl, 0.05% Tween 20), subsequently probed with scFv at 1:100 dilution at 4°C overnight, and developed via incubation with an HRP-labeled goat anti-mouse (IgG) secondary antibody (Abcam, Cambridge, UK) (1:5,000) at room temperature for 1 h. The membrane was visualized via exposure to X-ray film after the addition of a chemiluminescence substrate (Thermo, Waltham, MA, USA).

### Preparation of Aβ42 fibril

Based on a previous description [9] with minor modifications, Aβ42 fibrils were grown at a 0.2 mg/ml concentration in borate buffer (0.1 M boric acid, 2.5 mM NaCl, 2.5 mM sodium borate) at 37°C. Samples were taken at 24, 48, 72 and 96 h. In an Aβ42 fiber formation inhibition assay, 200 μl of a 0.2 mg/ml fibril solution was incubated with a 2-fold molar excess of scFv in borate buffer (pH 8.5) at 37°C, and the reaction was observed under transmission electron microscopy (TEM) at 0, 24, 48, 72 and 96 h. In the Aβ42 fiber disaggregation assay, mature Aβ42 fibers were incubated with a 2-fold molar excess of scFv in borate buffer (pH 8.5) at 37°C for 24 and 48 h, followed by analysis under TEM. In a dose-response assay, Aβ42 fibrils were incubated with different densities (1-fold, 5-fold and 10-fold) of scFv for 48, 72 and 96 h.

### Transmission electron microscopy

For this experiment, 20 μl of Aβ42 samples were added to 400-mesh carbon-coated nickel grids for 1 min and air-dried. Then, the samples on the nickel grids were stained with 1% (w/v) uranyl acetate for 1 min and air-dried. The specimens were observed under a JEM1400 transmission electron microscope (JEOL, Tokyo, Japan) at 80 kV [21]. The length of the fibrils was calculated using ImageJ 2x 2.1.4.7” [22].

### Thioflavin T binding assay

The inhibition of Aβ fibril formation by scFv was monitored via thioflavin T (ThT) fluorescence. In an Aβ42 fiber formation inhibition assay, 50 μM Aβ42 (in borate buffer, see “preparation of Aβ42 fibril”) was incubated with 250 μM A8 scFv, and the samples were removed for detection at different time points. In the Aβ42 fiber disaggregation assay, mature Aβ42 fibers were incubated with a 10-fold molar excess of scFv in borate buffer (pH 8.5) at 37°C for 24 and 48 h. The assay was performed with 20 μM ThT, 0.1 M boric acid, 2.5 mM NaCl, and 2.5 mM sodium borate. To block the binding of scFv, the anti-Aβ scFv was pre-incubated with Aβ1–11 and Aβ12–24 peptides in the Aβ aggregation and disaggregation assays, respectively. The fluorescence of the parallel reactions was measured with a Fluorescence Spectrophotometer F-7000 (Hitachi High-Tech, Japan) at 442 nm (Excitation) and 484.8 nm (Emission) with a scan speed of 240 nm/min. The assay was performed three times.

### Statistical analyses

The fibril length and ThT fluorescence data were analyzed using one-way or two-way ANOVAs and the chi-square test. An α level of 0.05 was used for all statistical significance tests.

## Results

### Expression, purification and identification of anti-Aβ scFv through rBacmid in baculovirus

To express the variable region of MAb A8 without the Fc fragment, the VL and VH genes were combined using the (G_4_S)_3_ linker sequence to form the VL-(G_4_S)_3_-VH or VH-(G_4_S)_3_-VL structures through a two-step SOE-PCR method (described in S1A Fig). Our data showed that the linker sequence was introduced by the primer after the first PCR step. The 300 bp of the VL-(G_4_S)_3_ (S1B Fig, lane 1), (G_4_S)_3_-VH (S1B Fig, lane 2), VH-(G_4_S)_3_ (S1B Fig, lane 3), and (G_4_S)_3_-VL genes (S1B Fig, lane 4) were amplified, which provided templates and primers for the following PCR. The 750 bp of the VL-(G_4_S)_3_-VH (S1C Fig, lane 1) and VH-(G_4_S)_3_-VL (S1C, lane 2) genes were produced as a result of the second PCR step.

To treat the Aβ aggregation system with the scFv expressed in eukaryotic cells, we attempted to construct a rBacmid encoding anti-Aβ scFv in the baculovirus expression system in which the viruses were packaged in Sf9 cells. According to our previous results from the *E*. *coli* system (data not shown), the expression level of VL-(G_4_S)_3_-VH was substantially higher than that of VH-(G_4_S)_3_-VL (data not shown); thus, the VL-(G_4_S)_3_-VH orientation was selected for subsequent expression in Sf9 cells. The scFv gene with the His tag at the N terminus (S2A Fig, lane 1) or at the C terminus (S2A Fig, lane 2) was produced via PCR and then cloned into a baculovirus package vector (pFastBac1). The recombinant plasmid pFastBac1-His-VL-(G_4_S)_3_-VH (S2B Fig, lane 1) or pFastBac1-VL-(G_4_S)_3_-VH-His (S2B Fig, lane 2) was identified via *Bam*HI/*Xho*I digestion. After transposition, the 3,000-bp M13 PCR products observed by agarose gel electrophoresis indicated that rBacmid-His-VL-(G_4_S)_3_-VH (S2C Fig, lane 1) and rBacmid-VL-(G_4_S)_3_-VH-His (S2C Fig lane 2) were correctly constructed.

**Fig 1:**
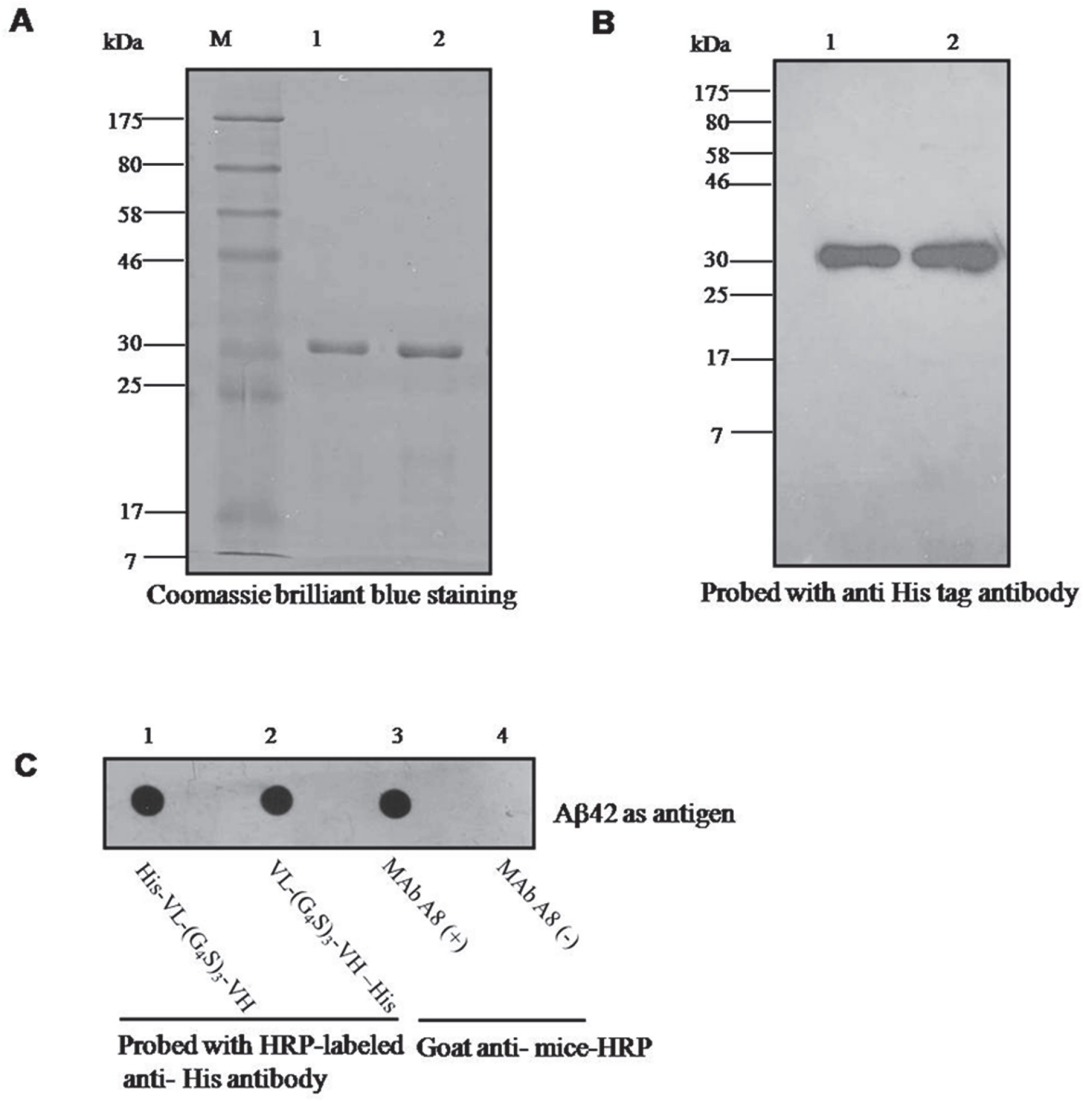
Immunoreactivity of anti-Aβ scFv expressed in baculovirus. (A) Coomassie brilliant blue staining in SDS-PAGE shows the purified His-VL-(G_4_S)_3_-VH (lane 1) and VL-(G_4_S)_3_-VH—His (lane 2) expressed in baculovirus with the correct molecular weight; (B) Western blot analysis with an anti-His tag antibody was used to identify the expression of scFvs described in (A); (C) Dot blot analysis shows the immunoreactivity of scFvs; the following antibodies were used: (1) His-VL-(G_4_S)_3_-VH, (2) VL-(G_4_S)_3_-VH—His, (3) monoclonal antibody A8 as a positive control, and (4) PBS as a negative control.

To identify the His-tagged scFv that was expressed in baculovirus, the proteins were first purified using a Ni-NTA affinity purification gel (see Materials and Methods). The Coomassie brilliant blue staining showed that purified His-scFv (lane 1 in Fig 1A or 1B) and scFv-His (lane 2 in Fig 1A or 1B) were expressed at 30 kDa based on SDS-PAGE (Fig 1A), which could be verified via Western blot analysis with an HRP-labeled anti-His antibody (Fig 1B). To confirm the expression of anti-Aβ scFv, the purified scFvs were analyzed using mass spectrometry (data not shown). The 750 bp of the VL-(G_4_S)_3_-VH (S1C Fig, lane 1) and VH-(G_4_S)_3_-VL (S1C Fig, lane 2) genes were also cloned into the pET-30a (+) vector and expressed in *E*. *coli* BL21 cells (data not shown).

### Immunoreactivity of anti-Aβ scFv expressed in baculovirus

To identify the bioactivity of the scFv expressed in baculovirus, the antigen was detected using dot blot and indirect ELISA analyses. The dot blot results showed that all of the scFvs expressed in baculovirus recognized Aβ42, with MAb A8 as the positive control and PBS as the negative control (Fig 1C); this was also confirmed via indirect ELISA (Table 3).

**Table 3:** The capacity of scFv to recognize Aβ42 through indirect ELISA.

Samples	A_450_ (Average)
His-VL-(G_4_S)_3_-VH	0.555
VL- (G_4_S)_3_-VH-His	0.387
A8 (+)	2.177
A8 (-)	0.054
Blank control	0.045

### The model of Aβ aggregation in the cell-free system

To establish a model of cell-free Aβ aggregation for the Aβ42 fiber formation inhibition and disaggregation assays, Aβ42 peptide was incubated in borate buffer at 37°C with various pH levels, and Aβ aggregation was detected using TEM at different time points. The morphology of the Aβ aggregates was nearly unstructured but nucleated, and fibrillogenesis was initiated before 24 h (S3A Fig); after 24 h, branched fibrils were detected (S3B Fig), and after a 48-h incubation in boric acid buffer, longer and matured fibrils were observed (S3C Fig and S3D Fig).

### Inhibition of on-pathway Aβ aggregation in the early stage by anti-Aβ scFv from baculovirus in a dose-dependent manner

**Fig 2:**
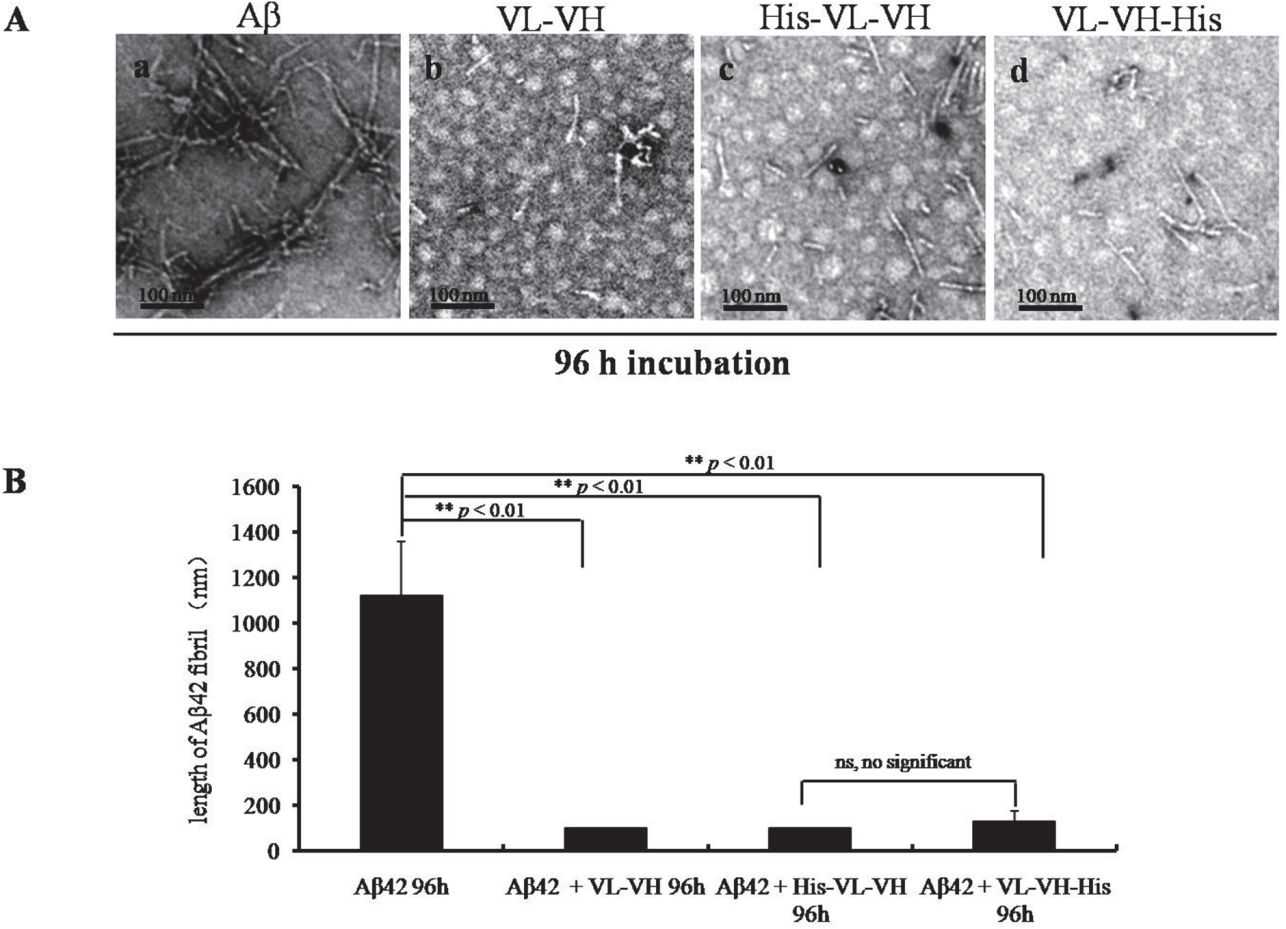
Inhibition of Aβ aggregation via the addition of an anti-Aβ scFv to the assembly reaction. TEM image of fibrils formed in the absence (a) or presence (b, c, d) of anti-Aβ scFvs (100 μM). (A) Aβ42 was incubated with (a) boric acid buffer control, (b) VL-(G_4_S)_3_-VH from *E*. *coli*, (c) His-VL-(G_4_S)_3_-VH, or (d) VL-(G_4_S)_3_-VH-His from baculovirus, for 96 h (scale bar = 100 nm); (B) The diagram shows the length of the fibrils in each group in (A). n = 5 independent experiments. Values are mean ± SEM. ns: not significant, **: p<0.001.

To determine whether anti-Aβ scFv expressed in baculovirus inhibits Aβ aggregation, the scFvs (100 μM) were added to the Aβ assembly reaction mixture at the start of Aβ aggregation. While long and branched fibrils were found in the control group incubated with boric acid buffer (Fig 2A a), the branched fibrils disappeared and the length of the fibrils decreased significantly in the scFv-treated group (Fig 2A b, c, and d) at 48 h (data not shown) and 96 h (Fig 2A and Fig 2B).

**Fig 3:**
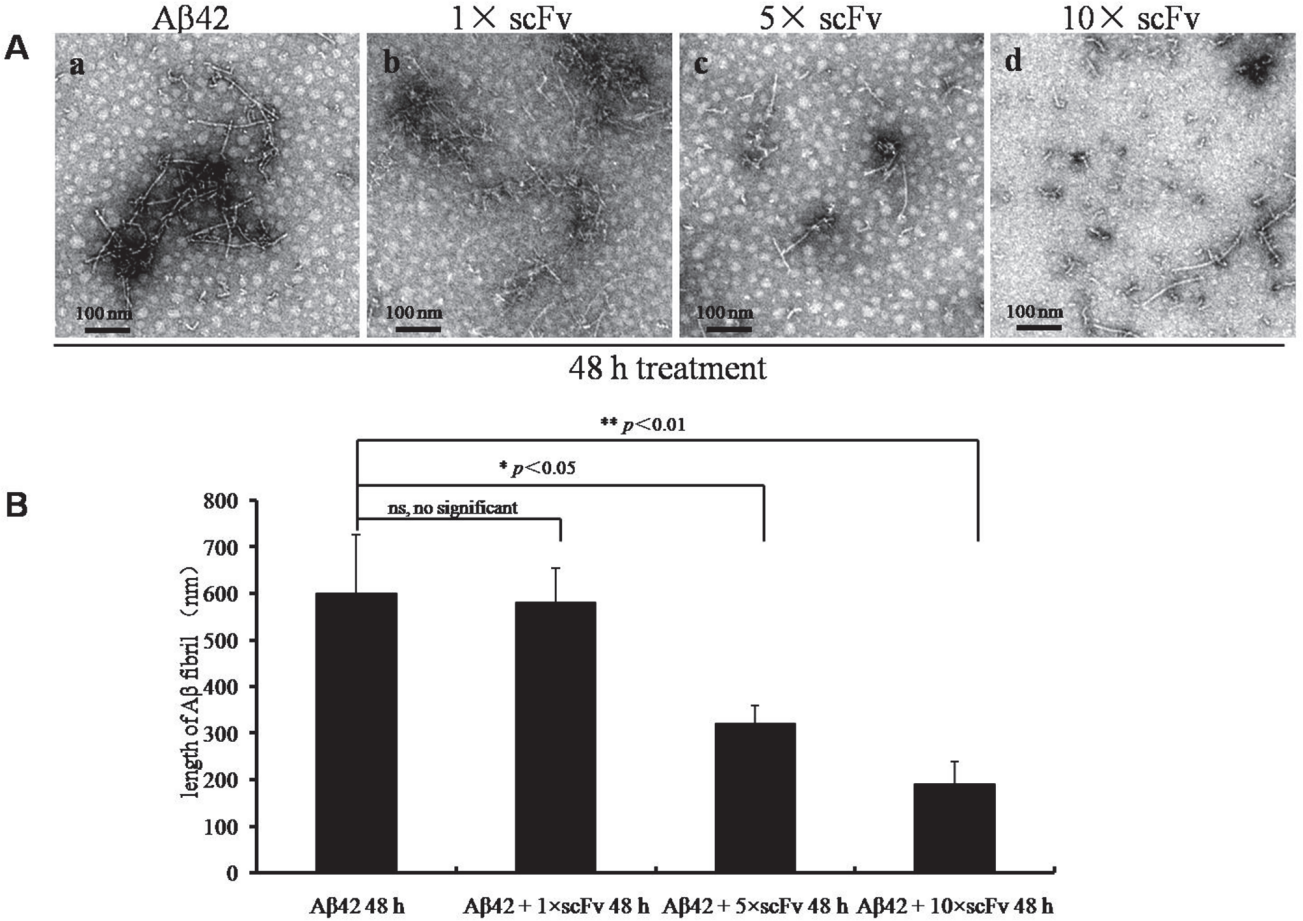
The dose-dependent increase in the anti-Aβ scFv effect. (A) TEM images of Aβ fibrils formed in the absence (a) or presence (b, c, d) of scFv. The scFvs were used at different concentrations, and Aβ was incubated with equal (b), five-fold (c) or ten-fold (d) molar excesses of scFv in boric acid buffer for 48 h (scale bar = 100 nm); (B) The diagram shows the length of the fibrils in each group in (A). n = 5 independent experiments. Values are mean ± SEM. ns: not significant, *: p<0.05, **: p<0.001.

To confirm the effects of scFv inhibition on Aβ fibril elongation, the Aβ peptides were treated with scFv in a dose-dependent manner starting at 0 h. Forty-eight hours later, the length of the fibrils was not significantly reduced (*p* > 0.05, Fig 3A b) in the 1× molar scFv-treated group compared with the buffer-treated control fibers (Fig 3A a); however, in the 5× molar scFv-treated group, the length of the fibrils was significantly decreased (*p* < 0.05, Fig 3A c and Fig 3B). As expected, if the concentration of scFv increased to 10× molar concentration, the length was highly significantly reduced (*p* < 0.01, Fig 3A d and Fig 3B), and only short and less-branched fibrils and aggregates were observed compared with the control group (Fig 3A a).

### Disaggregation of matured Aβ fibrils

**Fig 4:**
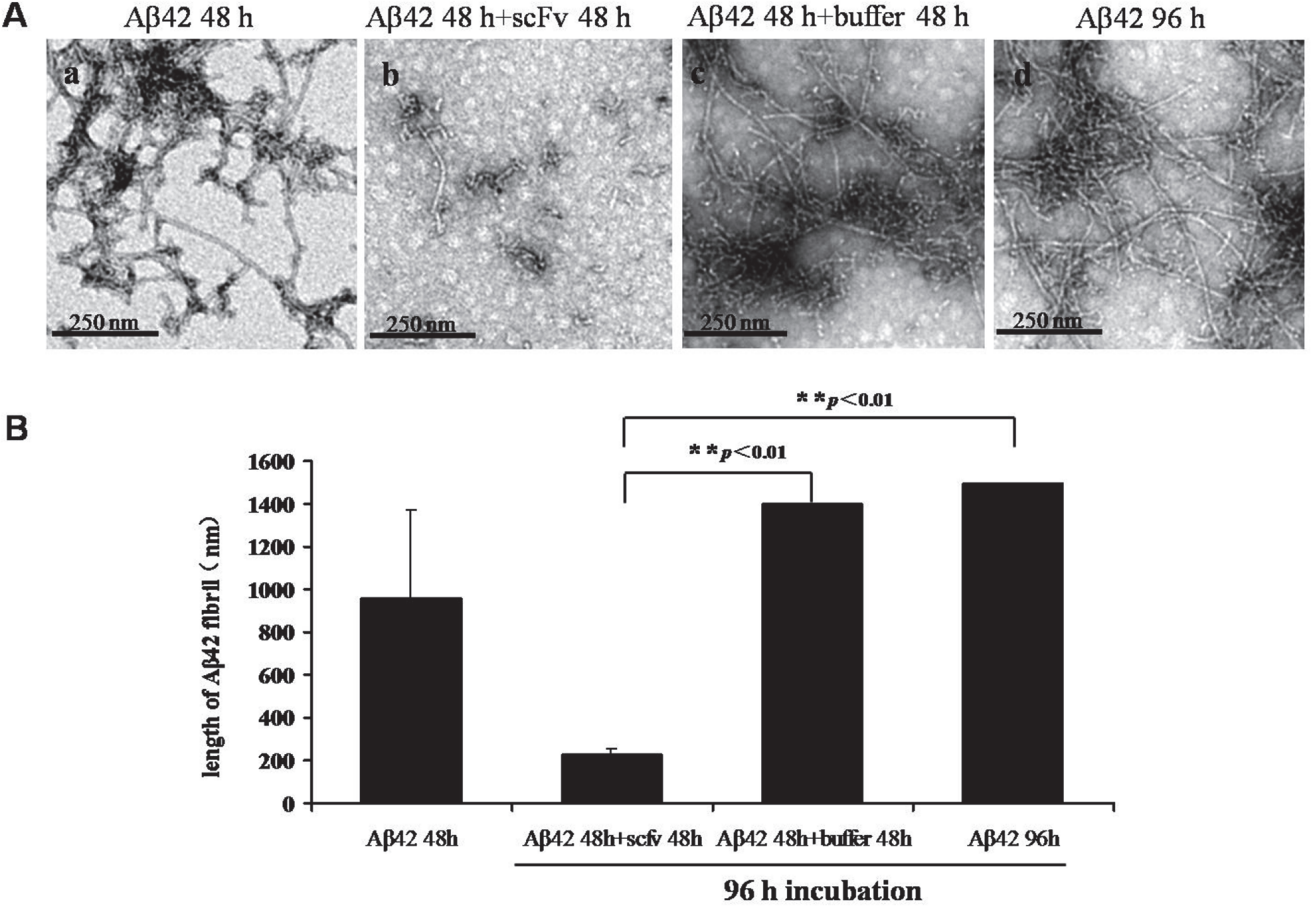
Disaggregation of mature Aβ fibrils (48 h incubation) by anti-Aβ scFv. (A) TEM images of the disaggregation of Aβ fibrils in the absence (a, c and d) or presence (b) of anti-Aβ scFv. (a) TEM image of A42 fibrils incubated in boric acid buffer for 48 h; (b) A42 fibrils from (a) were treated with the scFv [VL-(G_4_S)_3_-VH-His] for 48 h; (c) A42 fibrils from (a) were treated with scFv buffer for 48 h; (d) A42 from (a) was continuously incubated in boric acid buffer for a total of 96 h; (scale bar = 250 nm); (B) The histogram shows the corresponding lengths of the fibrils from each group mentioned in (A). **: p<0.01.

Based on previous studies [7], a forward direct reaction forms the fibrils from monomeric Aβ. Thus, we hypothesized that a reverse reaction from aggregated fibrils to oligomers or monomers may also exist. To test this hypothesis, we treated long, mature Aβ fibrils (grown for 48 h) with our scFv. We found that the scFv affected the kinetics of Aβ fibril formation, slowed the aggregation reaction and disaggregated the pre-formed fibrils (Fig 4A and 4B) into shorter and less branched fibrils. Our results suggested that anti-Aβ scFv treatment can result in both the inhibition of the aggregation and the enhancement of the disaggregation pathways of Aβ *in vitro*. To confirm these results, pre-formed long Aβ fibrils were treated with non-specific IgG and boiled in water, but the fibrils were not degraded (data not shown).

We also treated Aβ fibrils with the anti-Aβ scFv expressed in *E*. *coli* BL21 and obtained similar results. The number of Aβ fibrils decreased in both of the anti-Aβ scFv ([VH-(G_4_S)_3_-VL (S4C Fig and S4E Fig) and VL-(G_4_S)_3_-VH (S4D Fig and S4E Fig)])-treated groups (S4 Fig).

We have developed two orientations of scFvs in the *E*. *coli* system (Table 2), and the VL-(G_4_S_3_)-VH version exhibited a higher expression level. The VL-(G_4_S_3_)-VH version with an N-terminal or C-terminal His-tag was simultaneously expressed in baculovirus. The versions expressed in baculovirus have more pronounced effects on Aβ degradation, and the His-Tag does not interfere with the effects of scFvs.

### The effects of anti-Aβ scFv can be blocked by specific peptides

**Fig 5:**
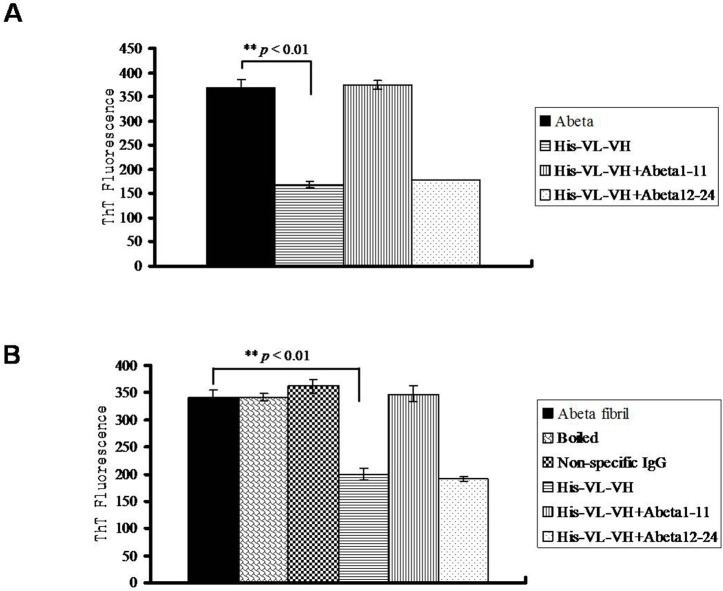
Blocking the effects of anti-Aβ scFv on Aβ aggregation and disaggregation by Aβ1–11 peptides. The anti-Aβ scFv [His-VL-(G_4_S)_3_-VH)] was incubated with Aβ1–11 and Aβ12–24 before being added to the Aβ aggregation and disaggregation reaction systems, as mentioned in Fig 2 and Fig 4. Thioflavin T (ThT) fluorescence was detected in the (A) Aβ aggregation assays and (B) Aβ disaggregation assays. **: p<0.01.

To confirm the TEM results further and to investigate the mechanisms of the anti-Aβ scFv regulation of on-pathway Aβ aggregation, we performed ThT binding assays and peptide blocking assays. Aβ-truncated peptides were used to block scFvs in the Aβ aggregation and disaggregation assays. The results showed that in the Aβ aggregation inhibition assays, anti-Aβ scFv reduced ThT fluorescence (*p* < 0.01), which in turn was blocked by pre-incubation of anti-Aβ scFv with Aβ1–11 but not Aβ12–24 (Fig 5A). In the Aβ disaggregation assays, our results showed that the ThT fluorescence was not reduced by non-specific IgG and heat denaturation (boiling in water) (Fig 5B). However, ThT fluorescence was reduced by incubation with A8 scFv (*p* < 0.01), which in turn could be blocked by Aβ1–11 but not by Aβ12–24 (Fig 5B).

## Discussion

The accumulation of Aβ deposits in amyloid plaques in the brain parenchyma is thought to play a critical role in the pathogenesis of AD [23]. Recent *in vitro* studies indicate that Aβ aggregation is responsible for triggering a cascade of physiological events that are critical for the initiation and progression of AD [8]. Additionally, our previous studies showed that an Aβ N-terminal targeting oligomer-selective MAb (A8) may have therapeutic potential [13]. Here, MAb A8-derived scFvs were expressed in baculovirus and used as regulators of on-pathway Aβ assembly. Based on our previous finding [13] that the full-length A8 IgG targeting amino acids 1–6 of Aβ reduced the Aβ load in the mouse brain, we tested whether and how an antibody fragment derived from A8 IgG retained this activity.

First, we constructed the scFv using a (G_4_S)_3_ linker to connect the variable regions, so that the final molecule consisted of a single polypeptide chain comprising an antibody heavy chain variable domain (VH), a flexible polypeptide linker, and a light chain variable domain (VL). Then, our data showed that this type of anti-Aβ scFv can be expressed solubly and purified under native conditions. Our results also demonstrated that the administration of A8 scFv inhibited Aβ aggregation. Furthermore, the A8 scFv was shown to disaggregate pre-formed fibrils. Based on these findings, an IgG scFv that lacks the Fc effector domain has evolved to represent a novel and promising therapeutic tool for the treatment of AD. The molecular weight of the scFv is lower than 30 kDa, which may facilitate its transfer across the blood-brain barrier [24]. Therefore, the advantage of elevated concentrations of scFv in the brain may increase the inhibition of Aβ aggregation/elongation and the promotion of Aβ degradation. Clearly, future *in vivo* studies of A8 scFv are necessary.

In this study, baculovirus was used to express A8 scFv due to its advantage of post-translational modification. The Bac-to-Bac Baculovirus Expression System provides the proper conditions for the modification of proteins after translation and disulfide bond formation [25], which is important to form the correct scFv interchain secondary structure. No long fibrils were detected if the baculovirus-produced A8 scFv was used at either the beginning or late stage of the assembly process (Fig 2 and Fig 4). These data indicate that baculovirus-expressed A8 scFv inhibited fibril formation and disaggregated the pre-formed fibrils, suggesting that baculovirus-expressed A8 scFvs participates in both the forward and reverse reactions of Aβ fibril formation.

We also investigated another Aβ N-terminal targeting antibody (A6F7, established by our group) in the Aβ aggregation and disaggregation assays with similar results (data not shown). However, the pre-formed long Aβ fibrils were not disassociated by the C-terminal targeting MAb (2D7, established by our group) (data not shown). The epitope of the A8 scFv is located at N-terminal amino acids 1–6 [14] of Aβ, which is not the domain that initiates the nucleation of Aβ fibrils. It is poorly understood why the N-terminus targeting scFv can influence C-terminus-related Aβ aggregation. However, this suggests that intermolecular interactions between Aβ42 N termini may be a therapeutic target via the regulation of C-terminal interactions that are necessary for fibril formation; this has been indicated in previous reports [7].

As described in detail previously [1], the failure in Aβ immunotherapy may also provide insights [1] for the mechanism study. Recently, phase III clinical trials of two monoclonal antibodies against Aβ (bapineuzumab and solanezumab) also failed to significantly improve clinical outcomes in patients with mild to moderate AD [26,27]. In this regard, antibodies with robust evidence of Aβ removal in specific stage of AD should be used for future clinical trials. Drugs that inhibit Aβ aggregation and degrade its fibrils should provide effects both in the early and late stages of AD. The former can be used to prevent disease, while the latter may be preferential for treatment of patients with clinical symptoms. In recent reports [1], one of the reasons proposed for the failure of antibody-based AD immunotherapy is that these antibodies target defects in mid- or late-AD. If anti-Aβ scFv significantly degrades fibers in the late stages without causing inflammation, it could be a promising candidate in new immunotherapy studies.

The IgG Fc fragment has been deleted in scFv. This presents an advantage in treatment by avoiding the adverse side effects associated with immunotherapy. Fc-mediated phagocytosis is one potential mechanism of AD immunotherapy [12,28]; however, this mechanism induces meningoencephalitis [29] and cerebral microbleeds [30]. The administration of scFv should not induce inflammation because of the lack of Fc fragments. However, it is not known whether the interaction between the antigen epitope and the scFv binding site would be modified after the deletion of the Fc segment. Further studies on this finding are necessary.

This study could not determine whether the nucleation process is interrupted by scFv; different detection systems and methods may be required. Therefore, we plan to optimize the reagents and molecules for nucleation inhibition to investigate the mechanisms underlying the effects of scFv.

## Conclusion

Taken together, in this study, a scFv derived from an anti-Aβ MAb was successfully expressed in baculovirus and was shown to recognize Aβ42 and affect the regulation of the ultrastructural dynamics of on-pathway Aβ aggregation and disaggregation. The baculovirus-expressed A8 scFv exerted effect on both the aggregation of Aβ monomers into fibrils and the disassembly of fibrils into smaller molecules. Therefore, the A8 scFv could be used independently or as a targeted fusion protein in AD therapeutic studies. The present study provided valuable insight into the regulation of the ultrastructural dynamics of Aβ aggregation and the development of therapeutic strategies for AD.

## Supporting Information

S1 Fig Construction of the anti-Aβ scFv gene fragments through gene splicing via overlap extension PCR (SOE PCR). (A) Diagram of the two-step SOE PCR process showing the connection of the VL and VH regions using a (G_4_S)_3_ linker; (B) Agarose gel electrophoresis was used to confirm the production of the 300-bp first-step PCR products, including VL-(G_4_S)_3_ in lane 1, (G_4_S)_3_-VH in lane 2, VH-(G_4_S)_3_ in lane 3, and (G_4_S)_3_-VL gene in lane 4. (C) Agarose gel electrophoresis was used to confirm the production of the 750-bp second-step PCR products, including VL-(G_4_S)_3_-VH in lane 1 and VH-(G_4_S)_3_-VL in lane 2.
(TIF)

S2 Fig Construction of the rBacmid encoding anti-Aβ scFv. (A) Agarose gel electrophoresis shows the His-VL-(G_4_S)_3_-VH (lane 1) and VL-(G_4_S)_3_-VH—His (lane 2) gene fragments containing *Bam*HI/*Xho*I sites (750 bp) for cloning into pFastBac1; (B) Agarose gel electrophoresis shows that the pFastBac1-His-VL-(G_4_S)_3_-VH (lane 1) and pFastBac1-VL-(G_4_S)_3_-VH-His (lane 2) constructs were identified by restriction endonuclease (*Bam*HI/*Xho*I) digestion (the 750-bp bands are the scFv genes cut from the vectors); (C) Agarose gel electrophoresis shows that the rBacmids were correctly constructed according to the 3,000-bp PCR band.
(TIF)

S3 Fig TEM images of Aβ aggregates at different time points. Aβ peptides were incubated in boric acid buffer and examined using TEM at 0, 24, 48, and 72 h after negative staining. TEM images of Aβ fibrils formed at the following different time points: (A) 0, (B) 24, (C) 48, and (D) 72 h.
(TIF)

S4 Fig The elongation of Aβ fibrils was inhibited by the anti-Aβ scFv expressed in *E*. *coli* BL21. (A) TEM images of Aβ aggregation at 0 h; (B) Aβ fibrils formed and elongated during 48 h of incubation in boric acid saline buffer; (C) and (D) The increase in the number of fibrils was inhibited by anti-Aβ scFv (VH-(G_4_S)_3_-VL or VL-(G_4_S)_3_-VH from *E*. *coli*) treatment for 48 h; (E) The diagram shows the number of fibrils longer than 200 nm in each group in (A), (B), (C) and (D). VL-VH indicates VL-(G_4_S)_3_-VH, and VH-VL indicates VH-(G_4_S)_3_-VL. ns: not significant. *: p<0.05, **: p<0.01.
(TIF)
