# Supplementary figures and images for: Single Chain Variable Fragment Against Aβ Expressed in Baculovirus Inhibits Abeta Fibril Elongation and Promotes its Disaggregation
Source: PLoS One. 2015 Apr 28;10(4):e0124736. doi: 10.1371/journal.pone.0124736 (PMC4412524; doi:10.1371/journal.pone.0124736)

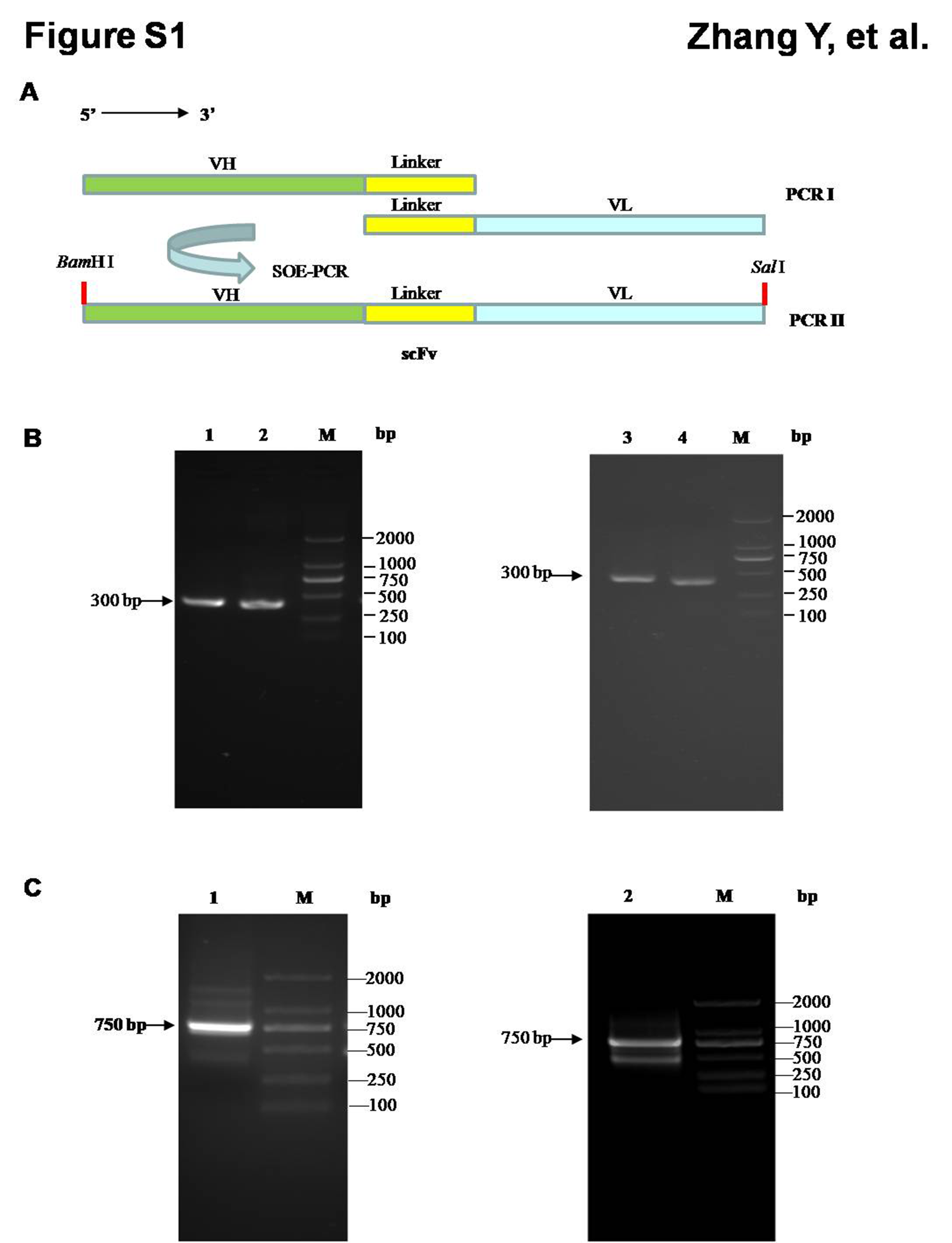

Supplement: S1 Fig — (A) Diagram of the two-step SOE PCR process showing the connection of the VL and VH regions using a (G4S)3 linker; (B) Agarose gel electrophoresis was used to confirm the production of the 300-bp first-step PCR products, including VL-(G4S)3 in lane 1, (G4S)3-VH in lane 2, VH-(G4S)3 in lane 3, and (G4S)3-VL gene in lane 4. (C) Agarose gel electrophoresis was used to confirm the production of the 750-bp second-step PCR products, including VL-(G4S)3-VH in lane 1 and VH-(G4S)3-VL in lane 2. (TIF) [file pone.0124736.s001.tif]

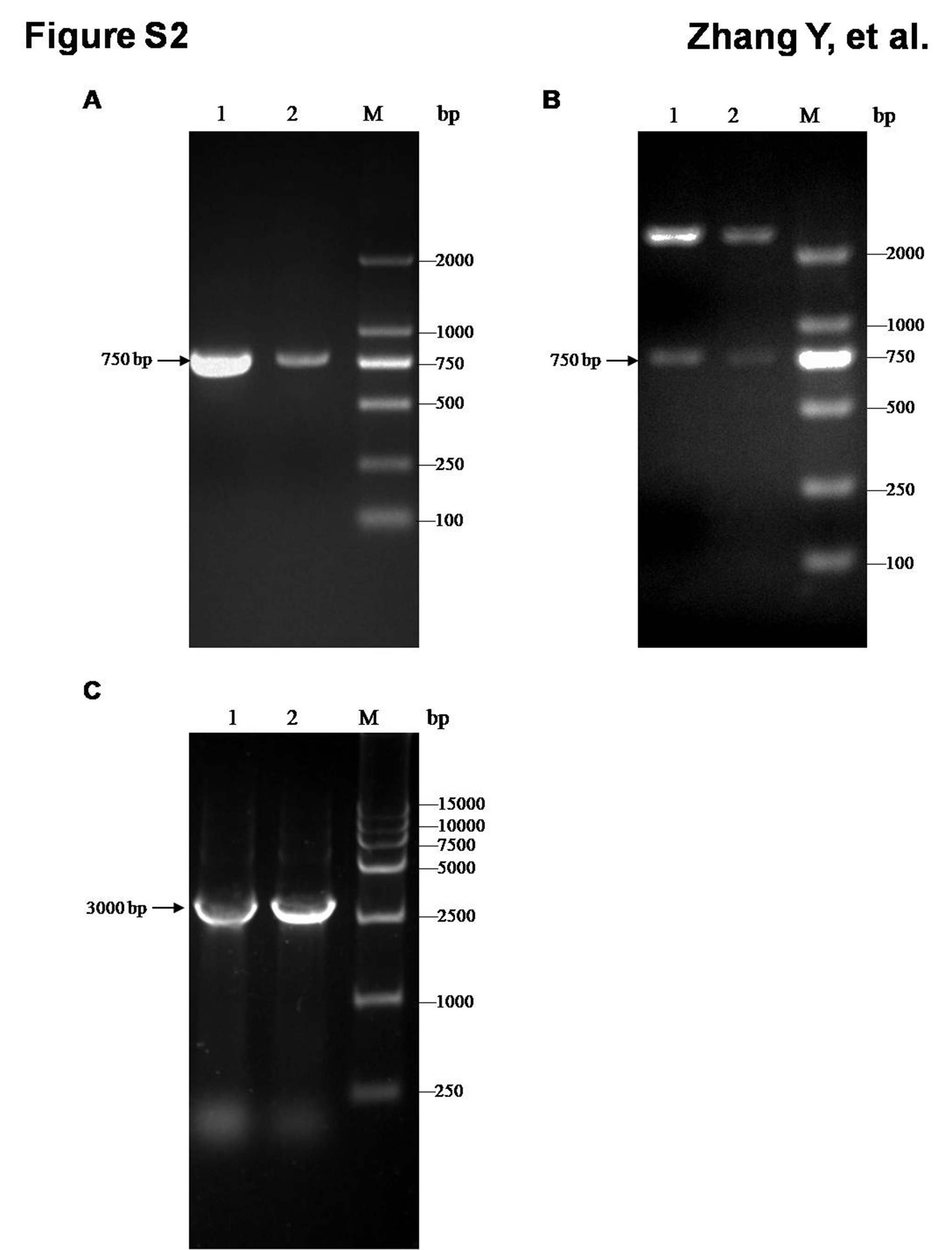

Supplement: S2 Fig — (A) Agarose gel electrophoresis shows the His-VL-(G4S)3-VH (lane 1) and VL-(G4S)3-VH—His (lane 2) gene fragments containing BamHI/XhoI sites (750 bp) for cloning into pFastBac1; (B) Agarose gel electrophoresis shows that the pFastBac1-His-VL-(G4S)3-VH (lane 1) and pFastBac1-VL-(G4S)3-VH-His (lane 2) constructs were identified by restriction endonuclease (BamHI/XhoI) digestion (the 750-bp bands are the scFv genes cut from the vectors); (C) Agarose gel electrophoresis shows that the rBacmids were correctly constructed according to the 3,000-bp PCR band. (TIF) [file pone.0124736.s002.tif]

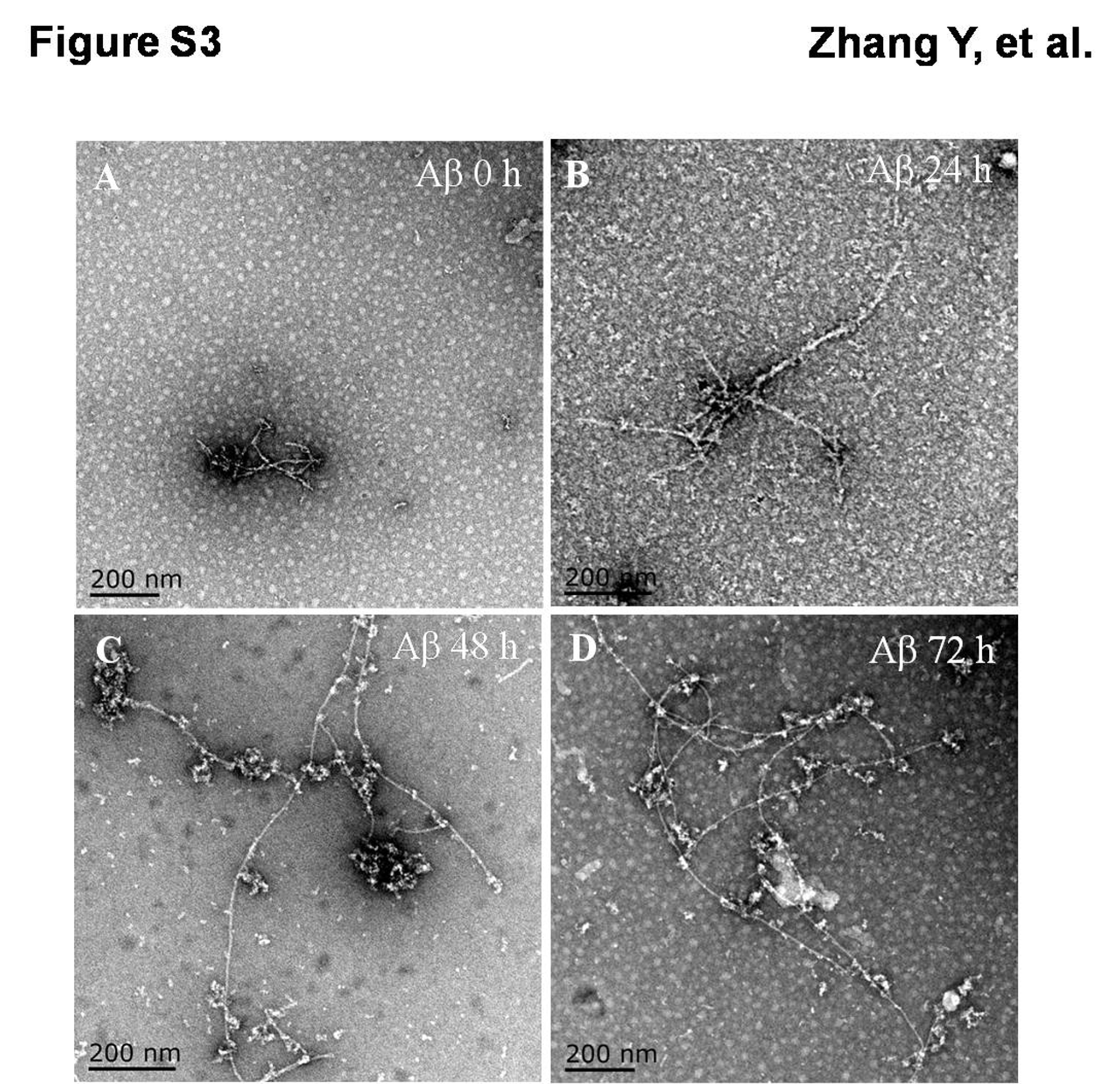

Supplement: S3 Fig — Aβ peptides were incubated in boric acid buffer and examined using TEM at 0, 24, 48, and 72 h after negative staining. TEM images of Aβ fibrils formed at the following different time points: (A) 0, (B) 24, (C) 48, and (D) 72 h. (TIF) [file pone.0124736.s003.tif]

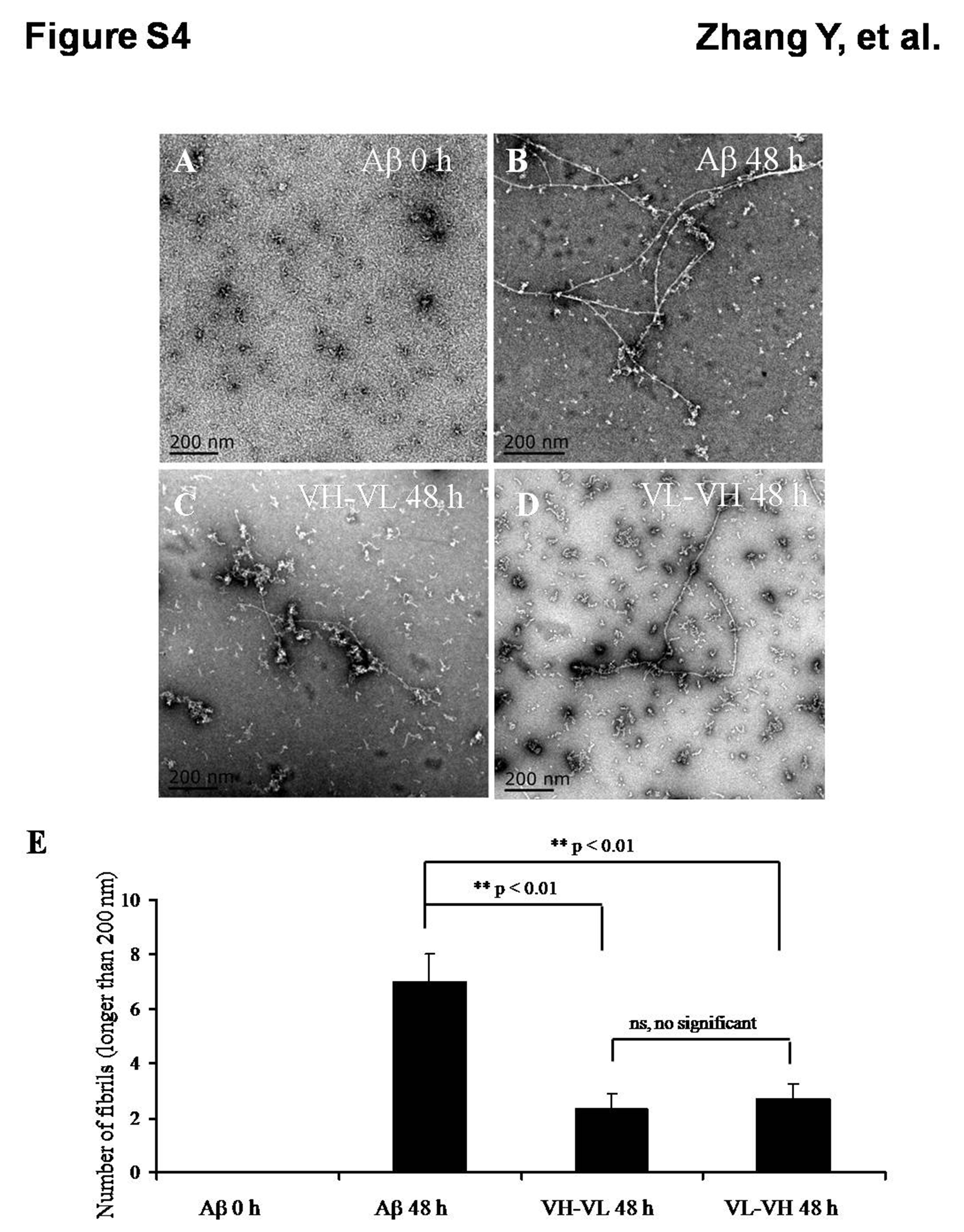

Supplement: S4 Fig — (A) TEM images of Aβ aggregation at 0 h; (B) Aβ fibrils formed and elongated during 48 h of incubation in boric acid saline buffer; (C) and (D) The increase in the number of fibrils was inhibited by anti-Aβ scFv (VH-(G4S)3-VL or VL-(G4S)3-VH from E. coli) treatment for 48 h; (E) The diagram shows the number of fibrils longer than 200 nm in each group in (A), (B), (C) and (D). VL-VH indicates VL-(G4S)3-VH, and VH-VL indicates VH-(G4S)3-VL. ns: not significant. *: p<0.05, **: p<0.01. (TIF) [file pone.0124736.s004.tif]
